# 3D-image-guided HDR-brachytherapy versus 2D HDR - brachytherapy after external beam radiotherapy for early T-stage nasopharyngeal carcinoma

**DOI:** 10.1186/1471-2407-14-894

**Published:** 2014-11-29

**Authors:** YuFeng Ren, QuanCheng Zhao, Hui Liu, YingJuan Huang, ZhenYu Wang, XinPing Cao, Bin S Teh, BiXiu Wen

**Affiliations:** Department of Radiation Oncology, The First Affiliated Hospital, Sun Yat-sen University, Guangzhou, 510080 P.R.China; State Key Laboratory of Oncology in Southern China, Department of Radiation Oncology, Cancer Center, Sun Yat-sen University, Guangzhou, 510060 P.R.China; Department of Neurosurgery, Binzhou People’s hospital, Binzhou, P.R.China; Department of Traditional Chinese medicine, The First Affiliated Hospital, Sun Yat-sen University, Guangzhou, 510080 P.R.China; Department of Radiation Oncology, the Methodist Hospital, 6565 Fannin, Houston, Texas 77030 USA

**Keywords:** Nasopharyngeal carcinoma, Radiotherapy, 3D-image-guided, Brachytherapy, Local control

## Abstract

**Background:**

Two-dimensional high-dose-rate brachytherapy (2D-HDR-BT) is an effective method of dose escalation for local tumor control in early T-stage nasopharyngeal carcinoma (NPC). Treatment outcomes for 3D-image-guided high-dose-rate brachytherapy (3D-image-guided-HDR-BT) after external beam radiotherapy (ERT) have not been examined in early T-stage NPC patients. The current study was designed to evaluate whether addition of 3D-HDR-BT to ERT showed further improvement in treatment outcomes in patients with early T-stage NPC when compared to 2D-HDR-BT after ERT.

**Methods:**

The current study retrospectively analyzed and compared treatment outcomes for patients with nonmetastatic stage T1-2b NPC treated with 2D-HDR-BT (n =101) or 3D-HDR-BT (n =118) after ERT. Patients in both groups were treated with ERT at a mean dose of 60 Gy and a brachytherapy dose of 12Gy (8 ~ 20Gy), 2.5 ~ 5Gy per fraction under local anesthesia.

**Results:**

Compared to patients treated with 2D-HDR-BT after ERT, patients treated with 3D-HDR-BT after ERT showed improvement in five-year actuarial local control survival rates (p = 0.024), local/regional relapse-free survival rates (p = 0.038), and disease-free survival rates (p = 0.021). Multivariate analysis showed that NPC patients treated with 3D-HDR-BT had improved local control survival (p = 0.042). The incidence rates of acute or chronic complications were similar between two groups.

**Conclusions:**

The current study showed that 3D-image-guided HDR-BT after ERT was an effective treatment modality for patients with stage T1-2 NPC with acceptable complications. The improvement in local tumor control and disease free survival is likely due to improved conformal dose distributions.

## Background

Nasopharyngeal carcinoma (NPC) is a radiosensitive disease, and radiotherapy is the standard therapy for non-disseminated NPC. Previous studies of radiotherapy treatment of NPC have reported good local control and overall survival rates [1, 2]. Teo et al. [3] have proposed a dose-tumor-control relationship above the conventional effective dose levels for early T-stage NPC patients.

Brachytherapy has been used to apply high doses of radiation directly to the primary tumor. Due to the steep nature of the rapid dose fall-off associated with the brachytherapy source, brachytherapy techniques can provide a higher nasopharyngeal tumor dose in comparison to the techniques of external beam therapy. Brachytherapy provides dose escalation for improving local tumor control and overall survival in early T-stage NPC patients [4, 5]. 3-D CT-based high-dose-rate brachytherapy (3D-HDR-BT) is a treatment method developed from 2D-HDR-BT as image-guided brachytherapy. This modern image-adpated 3D-HDR-BT can provide a higher conformal dose and higher precision distributions relative to 2D-HDR-BT. Previous studies have reported that 3D-HDR-BT after ERT treatment was effective in cervical cancer [6], breast cancer [7] and prostate cancer patients [8]. Ren et al. [9] reported 3D-image-guided CT-based HDR-BT achieved excellent local tumor control rate as salvage treatment to primary IMRT for patients with locally persistent NPC disease, especially for those with T1-2 disease at the initial diagnosis. However, no studies reported the use of 3D-HDR-BT after ERT for the treatment of stage I or II NPC.

In the current study, treatment outcomes of early T-stage NPC patients treated with ERT followed by 3D-HDR-BT or 2D-HDR-BT were analyzed retrospectively to evaluate the relative effectiveness of 3D-HDR-BT versus 2D-HDR-BT in NPC patients and to establish guidelines for future studies to determine whether improved conformality is associated with better therapeutic outcomes.

## Methods

### Patient populations and characteristics

A total of 118 patients with T1-2 stage NPC who were treated with 3D-HDR-BT after ERT at our center between May 2005 and January 2010 were enrolled in Group A. To evaluate the efficacy of this new technique relative to 2D-HDR-BT, 101 patients with T1-2 stage NPC who were treated with 2D-HDR-BT after ERT at our center between February 2003 and January 2005 were included in Group B. Eligible patients were aged 18–70 years with non-metastatic, histologically proven non-keratinising stage I or II nasopharyngeal carcinoma (6th AJCC). All participants had Karnofsky scores of at least 70, and adequate bone marrow, renal, and liver function. When combined, the male: female ratio of Groups A and B was 3:1, which included 162 males and 57 females. Histological examination showed that 97.7% of the patients in Groups A and B had World Health Organization (WHO) Type III disease, three patients had WHO Type I or II disease, and two patients had adenocarcinoma. The study was performed in accordance with the *Declaration of Helsinki* and was approved by the ethics committee of Sun Yat-Sen University Cancer Center. Written consent was given by the patients for their information to be stored in the hospital database, and we obtained consent to publish figures from the patient involved in this study.

### Clinical staging

All patients were diagnosed with biopsy-confirmed primary NPC. The extent of the disease was evaluated by thorough physical examination, direct fiber-optic examination, complete blood count, blood chemistry test, chest X-ray, bone scan, and magnetic resonance imaging (MRI)/computed tomography (CT) scans of the nasopharynx and neck. NPC disease progression was monitored carefully in each patient during the course of the study.

All MRI/CT materials and clinical records were reviewed to minimize heterogeneity in restaging. Two radiologists specializing in head-and-neck cancers evaluated all scans, and any disagreements were resolved by consensus. All patients were restaged according to the American Joint Committee on Cancer Staging System (6^th^ edition) [10]. All patients had nonmetastatic (M0) NPC. The T-stage distribution was determined for all NPC patients in Groups A and B. Group A consisted of 65 patients with stage T1 NPC, 34 patients with stage T2a NPC (nasal cavity tumors), and 19 patients with stage T2b NPC (parapharyngeal space extension). Group B consisted of 42 patients with stage T1 NPC, 41 patients with stage T2a NPC, and 18 patients with stage T2b NPC.

### Treatment methods

All patients were treated with definitive intent radiation therapy: the primary nasopharyngeal tumor received a mean dose of 60 Gy (range, 56 Gy to 62 Gy), followed by brachytherapy of 12Gy (8 ~ 20Gy), 2.5 ~ 5Gy per fraction. The external beam radiotherapy techniques have previously been reported [11]. During ERT, patients in both groups were evaluated fiber optically every week to assess tumor response to treatment.

#### Group A

**Figure 1:**
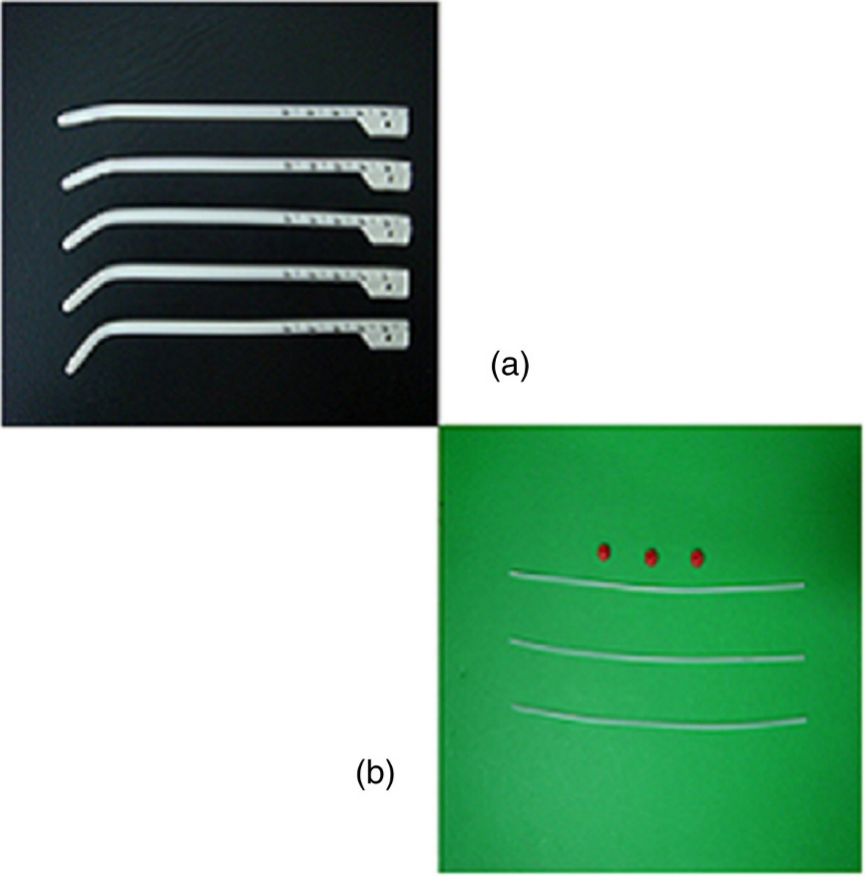
**Two nasopharyngeal applicators. (a)** Nasopharyngeal intracavitary brachytherapy applicator custom designed for T1-2a patients. **(b)** A ProGuide Needle (189.601 ProGuide Needle Set 6 F, sharp) was used as a nasopharyngeal applicator (microSelectron, Nucletron, nylon tube technique) for stage T2b NPC patients.

**Figure 2:**
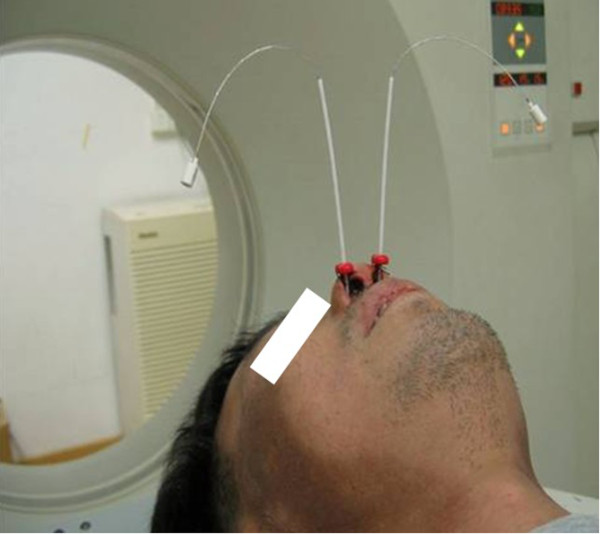
**Two customer designed applicators for nasopharyngeal brachytherapy were immobilized in the supine position.**

The 3D-HDR-BT for Group A was delivered after the completion of EBRT. All patients were treated with a high-dose-rate (HDR) afterloading machine (microSelectron, Nucletron, Veenendaal, the Netherlands) using a ^192^Ir source. For T1-2a patients, a custom designed nasopharyngeal brachytherapy applicators 10-90°tube set (Figure 1a) was positioned under local anesthesia with fiberoptic endoscopic guidance via the inferior meatus to the treatment locations. Patients were immobilized externally by a head-frame. For T2b patients, a ProGuide Needle was used as a nasopharyngeal applicator (microSelectron, Nucletron, nylon tube technique) (Figure 1b). The interstitial portion of the implant consisted of two to four stationary ProGuide Sharp Needles and was placed in the parapharyngeal tissues of the primary lesion. Treatment with 3D-HDR-BT was performed as follows: two to four applicators were placed at treatment positions near the primary lesion under local anesthesia with fiberoptic endoscopic guidance via the inferior meatus and the applicators were then immobilized, their correct position is verified by MRI and the procedure is generally well tolerated CT scanning of the nasopharyngeal region was performed (0.2 cm step and 0.2 cm slice thickness (Figure 2). The primary nasopharynx tumor received 60 Gy of EBRT, the neck region received an accumulated radiation dose of 50 Gy, while the involved areas of the neck received 60–62 Gy. For 3D-BT,the GTV was determined by a radiation oncologist as the macroscopic extent of the persistent disease area, the CTV included the persistent disease area and the nasopharyngeal primary tumor area. For patients with complete response, The GTV do not delineate, the CTV included the nasopharyngeal primary tumor area. All patients received 12Gy (8 ~ 20Gy), 2.5 ~ 5Gy per fraction over the entire implant volume

**Figure 3:**
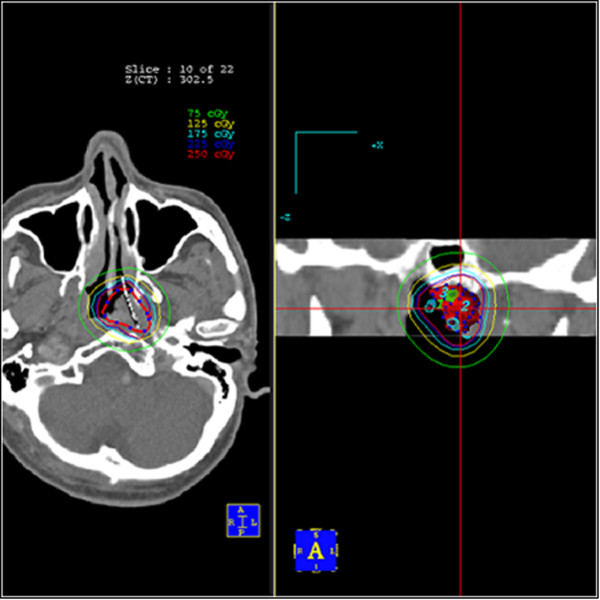
**Axial computed tomography (CT) image of a stage T1-2a NPC patient showing two brachytherapy intracavitary applicators** ***in situ*** **with 2.5 Gy, 2.25 Gy, 1.75 Gy, 1.25 Gy, and 0.75 Gy isodose lines**.

Following all target volumes defined and the applicators reconstruction, The PLATO PBS was used to calculate the dosimetry for a HDR ^192^Ir stepping source. The distance between each source step was 2.5 mm, the dose optimization was done step by step manually. This planning strategy enables us to build up the real time isodose distribution in all CT slices to increase accurate delivery of the prescribed dose, the isodose distribution can be calculated and demonstrated in coronal and sagittal planes, see Figure 3.

#### Group B

The 2D-HDR-BT for Group B was delivered after the completion of EBRT. The intracavitary brachytherapy applicators were positioned with fiberoptic endoscopic guidance via the inferior meatus to the treatment locations abutting on the posterosuperior wall of the nasopharynx. The patients were then immobilized externally by a head-frame. The separation between the central axis of the applicators ranged from 1.1 - 1.5 cm. The dose was prescribed at a distance of 1 cm from the center of the surface as defined by the sources.

#### Chemotherapy

Three cycles of adjuvant chemotherapy were given to patients in the concurrent chemoradiotherapy by intravenous infusion of 80 mg/m^2^ cisplatin on day 1 and 800 mg/m^2^ per day fluorouracil on days 1–5 (120 h infusion).

### Follow-up

All patients were seen and carefully monitored during follow-up with a radiation oncologist after the treatment schedule was completed. Acute and late complications were scored according to the criteria of the Radiation Therapy Oncology Group (RTOG) scoring system during each follow-up visit.

### Statistical analyses

The Statistical Package for Social Sciences (SPSS, Version 13.0, Chicago, IL) was used for statistical analyses. Comparisons of patient clinical-pathologic characteristics between Groups A and B were evaluated by the chi-square test. Survival rates were calculated from the radiotherapy start date. Estimates of the actuarial survival rate were determined by Kaplan-Meier estimate [12]. The log-rank test was used to compare survival curves [13]. Multivariate analysis of outcome data was performed using Cox regression analysis [14]. A p value of ≤0.05 was considered statistically significant. End points were defined as follows: local control survival (LCS), time to local recurrence; overall survival (OS), time to death from any cause; disease-free survival (DFS), time to disease recurrence, disease progression, metastasis, or death from disease-related causes; cause-specific survival (CSS), time to death due to disease; local/regional relapse-free survival (LRFS), time to recurrence/progression involving the primary site with or without regional involvement (deaths from local or regional recurrences were included in this definition); and distant metastasis-free survival (DMFS), time to distant metastatic recurrence, with or without prior or concurrent local/regional recurrence.

## Results

Between May 2005 and January 2010, a total of 118 patients with stage T1-2 NPC were treated with 3D-HDR-BT after ERT at our center were enrolled. 101 patients with stage T1-2 NPC were treated with 2D-HDR-BT after ERT between February 2003 and January 2005 in Group B. The median follow-up was 57 · 8 months (range 33.9–117 · 0). The two treatment groups were well balanced in terms of baseline demographic and clinical characteristics, and radiotherapy technique (Table 1).

**Table 1:** **Demographic and clinical characteristics of the two patient groups**

Variables	Group A	Group B	p-value
	3D-HDR-BT (n =118)	2D-HDR-BT (n =101)	
**Age (years)**			0.594
≤ 45	65 (11.9%)	52 (20.8%)	
> 45	53 (88.1%)	49 (79.2%)	
Mean	44.6	47.1	
Median	43	45	
Range	19 - 80	18 - 73	
**Gender**			0.100
Male	88 (74.6%)	65 (64.4%)	
Female	30 (25.4%)	36 (35.6%)	
**Histology**			0.529
WHO type I/II	2 (1.7%)	3 (3.0%)	
WHO type III	116 (98.3%)	98 (97.0%)	
**T-stage**			0.079
T1	65 (55.1%)	42 (41.6%)	
T2a	34 (28.8%)	41 (40.6%)	
T2b	19 (16.1%)	18 (17.8%)	
**N-stage**			0.740
N0-1	85 (72.0%)	74 (73.3%)	
N2-3	33 (28.0%)	27 (26.7%)	
**Chemotherapy**			0.740
Given	33 (28.0%)	27 (26.7%)	
Not given	85 (72.0%)	74 (73.3%)	
**EBRT modality**			
2D-CRT	96 (81.4%)	88 (87.1%)	0.662
3D-CRT	19 (16.1%)	13 (12.9%)	
IMRT	3 (2.5%)	0 (0%)	

*Abbreviations*: *2D-BT*, two-dimensional high-dose-rate brachytherapy; *3D-BT*, three-dimensional high-dose-rate brachytherapy; *WHO*: World Health Organization; 2D-CRT = two-dimensional radiotherapy. IMRT = intensity-modulated radiotherapy. 3D-CRT = three-dimensional conformal radiotherapy.

### Local and regional control

**Figure 4:**
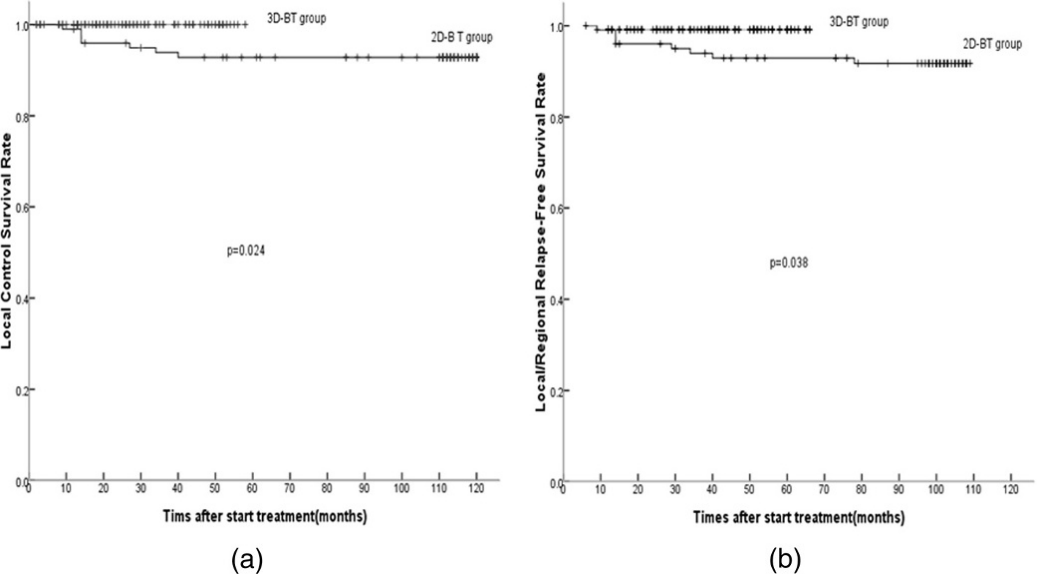
**Five-year actuarial survival curves for the 3D-HDR-BT group and the 2D-HDR-BT group. (a)** local control survival (LCS); **(b)** local/regional relapse-free survival (LRFS).

Local control was defined as no relapsed tumor within 6 months of completion of primary radiotherapy, and local recurrence, which was proven pathologically by biopsy to have local persistence, was failure beyond 6 months. Seven patients in Group B developed local recurrence beyond six months after the completion of the treatment schedule, and none of the patients in Group A developed local recurrence. The five-year actuarial LCS rates for Groups A and B were 100% and 93.1%, respectively (p = 0.024) (Figure 4a). One patient in Group A and one patient in Group B developed regional nodal-involved recurrence. The five-year actuarial LRFS rates in Groups A and B were 99.2% and 92.1% (Figure 4b), respectively; the LRFS curves for the two groups were statistically significant (p = 0.038).

### Survival rates

**Figure 5:**
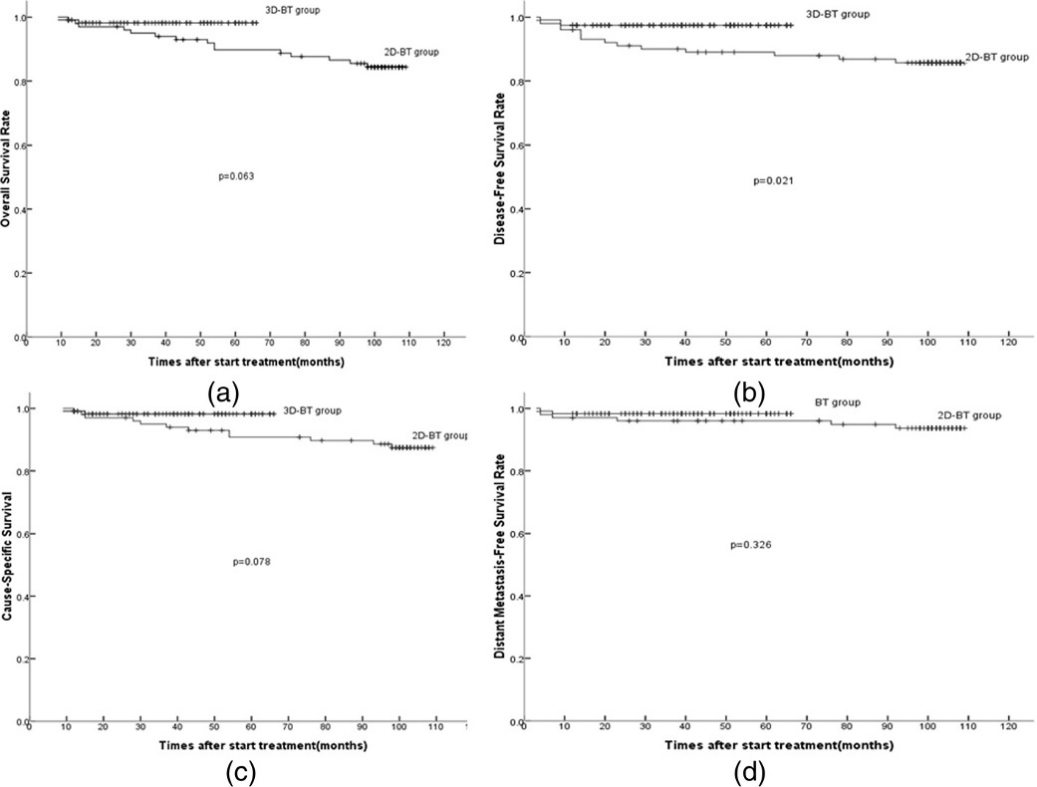
**Five-year actuarial survival curves. (a)** disease-free survival (DFS); **(b)** overall survival (OS); **(c)** cause-specific survival (CSS); and **(d)** distant metastasis-free survival (DMFS).

The five-year actuarial OS, DFS, CSS, and DMFS curves between the two groups were determined (Figure 5 a ~ d), and the corresponding rates for Groups A and B were 98.3% and 85.1% (OS, p = 0.063), 97.5% and 86.1% (DFS, p = 0.021), 98.3% and 88.1% (CSS, p = 0.078), as well as 98.3% and 94.1% (DMFS, p = 0.326), respectively. The DFS rates were statistically significant between the two groups. There was a trend toward improvements in other endpoints in Group A, but the difference was not statistically significant between the two groups.

### Multivariate analyses

All potential prognostic factors, including gender, age (≤45 years; >45 years), T-stage (T1, T2a, T2b), N-stage (0–3) and brachytherapy techniques (the administration of 3D-HDR-BT or 2D-HDR-BT) and EBRT techniques (2D-CRT, 3D-CRT & IMRT) were included in the Cox proportional hazards model by backward elimination of the insignificant explanatory variables. The results of multivariate analyses are summarized in Table 2. The Cox proportional hazards analysis showed that the T category was an unfavorable prognostic variable with regard to LCS, DFS, and LRFS endpoints. Multivariate analysis also showed that the N category was an unfavorable prognostic factor with regard to DFS, OS, CSS, and LRFS. Group A had a lower hazard of LCS than Group B (DFS = 0.232, 95% CI: 0.057 - 0.949, p = 0.042).

**Table 2:** **Summary of multivariate analysis of prognostic factors**

	Factors	B	p-value	Exp (B)	Exp (B) (95% CI)
**LCS**	T category	2.298	0.005	9.959	1.977 - 50.169
	N category	1.460	0.042	0.232	0.057 - 0.949
	3D-BT vs. 2D-BT	NS			
**DFS**	T category	0.973	0.031	2.647	1.093 - 6.407
	N category	2.950	< 0.001	19.113	5.621 - 64.997
	3D-BT vs. 2D-BT	NS			
**OS**	T category	NS			
	N category	2.625	< 0.001	13.800	4.128 - 46.127
	3D-BT vs. 2D-BT	NS			
**LRFS**	T category	1.407	0.025	4.083	1.192 - 13.989
	N category	NS			
	3D-BT vs. 2D-BT	NS			
**CSS**	T category	NS			
	N category	3.106	< 0.001	22.324	6.073 - 82.067
	3D-BT vs. 2D-BT	NS			
**DMFS**	T category	NS			
	N category	4.043	< 0.001	57.020	9.307 - 349.347
	3D-BT vs. 2D-BT	NS			

*Abbreviations*: *CI*, confidence interval; *2D-BT*, two-dimensional high-dose-rate brachytherapy; *3D-BT*, three-dimensional high-dose-rate brachytherapy; *NS*, not significant.

### Side effects and complications

Toxicity was acceptable for the patients in Groups A and B. Five patients experienced RTOG grade III-IV hematological toxicity; of those patients with N-stage (N2-3) NPC treated with concurrent chemoradiotherapy (DDP 40 mg/m^2^), three patients experienced grade-III mucositis. Complication rates were similar between Groups A and B when all patients were compared. The most common radiation-related complication was xerostomia. Almost all patients developed xerostomia via large parallel opposed fields. Slight xerocheilia were seen commonly, there was no severe xerocheilia. Total hearing loss, neck fibrosis limiting movement, severe epistaxis requiring blood transfusion, pituitary-endocrine dysfunction, and neurological complications (temporal lobe necrosis and cranial nerve palsy) were not seen in this series. No significant late toxicities were caused by brachytherapy. Complications associated with nasopharyngeal grade-II ulceration/necrosis were observed in three in Group A patients with foul-smelling crust and/or headache, and in two patients in Group B. A total of 19 patients with stage T2b disease experienced small amounts of blood loss in Group A; the average blood loss was approximately 5 cc during the course of the interstitial implantation and removal. All patients were managed with conservative treatment measures and the bleeding was always self-limiting.

## Discussion

Carcinoma of the nasopharynx has a propensity to local recurrence, and has always been a challenge to the radiotherapist [15–18].With advances in remote-controlled HDR after loading systems and the ease of applicators, the treatment plans can easily be carried out [19].

In the current study, the treatment outcomes of CT-based 3D HDR-BT versus 2D HDR-BT were evaluated in early T-stage NPC patients. CT-based 3D HDR-BT with stepping source provided good coverage of the target volume, and dose distribution optimization. Meanwhile, CT-based 3D treatment planning obtained excellent visualization of the persistent disease and normal structures. A 100% isodose line was selected to cover the entire target as optimally as possible, then manual optimization on each CT slice was done interactively by dragging the 100% isodose line to cover the target volume as conformally as possible in this study. Our experience shows that this CT-based-3D planning approach improved target volume delineation and optimal coverage, and is more accurate and much easier the conventional orthogonal film dosimetry. The follow-up results showed that 3D-HDR BT arm was an effective treatment modality for patients with stage T1-2 NPC with acceptable complications. We speculate the improvement in local tumor control and disease free survival is likely due to improved conformal dose distributions in 3D-HDR BT arms, or may be because of the short follow-up time.

### Improved NPC treatment outcomes with 3D-HDR-BT

The results of the current study showed that 3D-HDR-BT after EBRT was more effective in the treatment of T1-2 NPC than 2D-HDR-BT after EBRT, as shown by a statistically significant improvement in LCS and DFS. Conformality can be improved by using the CT-based 3D-HDR-BT treatment plan, and we hypothesized that improvements in LCS and DFS with 3D-HDR-BT were due to the conformal dose distributions [3, 20]. The dose distribution of 3D-HDR-BT can also cover the target for patients whose tumors are associated with the parapharygeal space better.

Retrospective analysis of the two groups showed that five patients with stage T2b disease who developed local recurrence were in the arm of 2D brachytherapy. The role of brachytherapy in patients with stage T2b disease is controversial [9, 21]. Levendag et al. [22] reported that some patients with stage T2b NPC lesions may benefit from dose-escalation by brachytherapy boost when the tumor had regressed to the clinical target volume after EBRT pretreatment. In the current study, when stage T2b NPC patients in Group A were implanted interstitially and the nylon afterloading tubes were placed in the parapharyngeal tissues of the primary tumor site, the 3D-HDR-BT led to enhanced conformal optimization. Local tumor control may be improved by 3D-HDR-BT in patients initially diagnosed with T2b disease, which is in accordance to the report by Rosenblatt E et al. [23] that the addition of an imaging guided brachytherapy boost to external beam radiotherapy significantly improved local tumor control in patients with T1-2 nasopharyngeal carcinoma. None of the Group A patients with stage T2b NPC developed local recurrence, although five Group B patients with stage T2b NPC relapsed.

### Unimproved NPC treatment outcomes with 3D-HDR-BT

Despite showing improvements in LCS and DFS, 3D-HDR-BT treatment did not improve the distant control rates of all N category tumors. These results are consistent with a previous study by Leibel SA *et al.*[24], which showed that nasopharyngeal tumors have a higher probability of micrometastatic dissemination at initial diagnosis, and concluded that the effect of local tumor control on survival cannot be determined without more effective methods to treat disseminated disease.

To determine the effect of local tumor control on distant tumor control, we should detect distant metastases as soon as possible after the initial diagnosis. A study by Chang JT *et al.*[25] used 18 F-fluorodeoxyglucose positron emission tomography (FDG-PET) and found that 11% of distant metastases in NPC patients were not discovered using the conventional staging workup (CWU). Based on this finding, they suggested that FDG-PET diagnosed stage M NPC disease more accurately and sensitively than CWU. However, the cost of FDG-PET was so high that it was not performed for each patient. Therefore, it was uncertain whether improved local tumor control by 3D-HDR-BT was associated with improved distant tumor control.

### Complications

In the current study, the side effects associated with 3D-HDR-BT after EBRT were less severe than some of the toxicity reports associated with conventional dose levels of EBRT alone [26]. Due to the rapid dose fall-off of the radiotherapy source, brachytherapy can deliver a high radiation dose to the parapharyngeal tumor region while delivering a low radiation dose to the pituitary fossa, the optic nerve/chiasm, the temporal lobes, the temporomandibular joint, and the middle ear. Therefore, severe complications were not commonly observed, and the complication rates were similar for the 3D-HDR-BT and 2D-HDR-BT treatment groups. Both 3D-HDR-BT and 2D-HDR-BT treatment modalities can provide highly conformal dose distribution and improved normal tissue sparing. Patients in the 3D-HDR-BT and 2D-HDR-BT groups did not show significant differences in the acute or chronic incidence rates of radiation complications. Mild chronic radiation nasopharyngeal ulceration/necrosis was seen in both groups, which included a foul-smelling crust and/or headache. Many patients experienced xerostomia as a long-term side effect, which may not be due to the brachytherapy. During the long-term follow-up, the only minor complication related to brachytherapy was synechiae of the nasal mucosal linings, which can be treated easily. In future treatment plans, synechiae of the nasal mucosal linings can be reduced using these measures. In the current study, patient mortality was not related to complications.

The interstitial implantation procedure was well tolerated by all stage T2b NPC patients in Group A. Some patients experienced small amounts of blood loss during the course of interstitial implantation. Bleeding was managed with conservative treatment measures and was always self-limiting: no patients required blood transfusions due to excessive bleeding during the course of treatment.

## Conclusions

External beam radiation therapy combined with interstitial/intracavitary 3D-image-guided HDR-brachytherapy showed improved local tumor control relative to traditional 2D-HDR-BT with acceptable toxicity levels. The use of 3D-image-guided HDR-BT for stage T2b NPC patients is relatively complex but well tolerated, the administration of interstitial 3D-HDR-BT achieved excellent local control. Improved therapeutic benefits associated 3D-image-guided HDR-BT may be due to enhanced target volume coverage and improved conformality. Prospective, randomized studies should be conducted to further evaluate the therapeutic benefits of 3D-image-guided HDR-brachytherapy for NPC patients.

## References

[1] Wu F, Wang R, Lu H, Wei B, Feng G, Li G, Liu M, Yan H, Zhu J, Zhang Y, Hu K (2014). Concurrent chemoradiotherapy in locoregionally advanced nasopharyngeal carcinoma: treatment outcomes of a prospective, multicentric clinical study. Radiother Oncol.

[2] Wang WY, Twu CW, Chen HH, Wang WY, Twu CW, Chen HH, Jiang RS, Wu CT, Liang KL, Shih YT, Chen CC, Lin PJ, Liu YC, Lin JC (2013). Long-term survival analysis of nasopharyngeal carcinoma by plasma Epstein-Barr virus DNA levels. Cancer.

[3] Teo PM, Leung SF, Lee WY, Zee B (2000). Intracavitary brachytherapy significantly enhances local control of early T-stage nasopharyngeal carcinoma: the existence of a dose-tumor-control relationship above conventional tumoricidal dose. Int J Radiat Oncol Biol Phys.

[4] Leung TW, Wong VY, Sze WK, Lui CM, Tung SY (2008). High-dose-rate intracavitary brachytherapy boost for early T stage nasopharyngeal carcinoma. Int J Radiat Oncol Biol Phys.

[5] Levendag PC, Keskin-Cambay F, de Pan C, Idzes M, Wildeman MA, Noever I, Kolkman-Deurloo IK, Al-Mamgani A, El-Gantiry M, Rosenblatt E, Teguh DN (2013). Local control in advanced cancer of the nasopharynx: is a boost dose by endocavitary brachytherapy of prognostic significance?. Brachytherapy.

[6] Madan R, Pathy S, Subramani V, Sharma S, Mohanti BK, Chander S, Thulkar S, Kumar L, Dadhwal V (2014). Comparative evaluation of two-dimensional radiography and three dimensional computed tomography based dose-volume parameters for high-dose-rate intracavitary brachytherapy of cervical cancer: a prospective study. Asian Pac J Cancer Prev.

[7] Oden J, Toma-Dasu I, Yu CX, Feigenberg SJ, Regine WF, Mutaf YD (2013). Dosimetric comparison between intra-cavitary breast brachytherapy techniques for accelerated partial breast irradiation and a novel stereotactic radiotherapy device for breast cancer: GammaPod. Phys Med Biol.

[8] Marina O, Gustafson GS, Kestin LL, Brabbins DS, Chen PY, Ye H, Martinez AA, Ghilezan MI, Wallace M, Krauss DJ (2014). Comparison of dose-escalated, image-guided radiotherapy vs. dose-escalated, high-dose-rate brachytherapy boost in a modern cohort of intermediate-risk prostate cancer patients. Brachytherapy.

[9] Ren YF, Cao XP, Xu J, Ye WJ, Gao YH, Teh BS, Wen BX (2013). 3D-image-guided high-dose-rate intracavitary brachytherapy for salvage treatment of locally persistent nasopharyngeal carcinoma. Radiat Oncol.

[10] Greene FL, Page DL, Fleming ID, Al. E (2002). AJCC cancer staging handbook from the AJCC cancer staging manual.

[11] Lei C, Chao-Su H, Chen L, Hu CS, Chen XZ, Hu GQ, Cheng ZB, Sun Y, Li WX, Chen YY, Xie FY, Liang SB, Chen Y, Xu TT, Li B, Long GX, Wang SY, Zheng BM, Guo Y, Sun Y, Mao YP, Tang LL, Chen YM, Liu MZ, Ma J (2012). Concurrent chemoradiotherapy plus adjuvant chemotherapy versus concurrent chemoradiotherapy alone in patients with locoregionally advanced nasopharyngeal carcinoma: a phase 3 multicentre randomised controlled trial. Lancet Oncol.

[12] Kaplan EL, Meier P (1958). Nonparametric estimation from incomplete observations. J Am Stat Assoc.

[13] Peto R, Pike MC, Armitage P, Et A (1958). Design and analysis of randomized clinical trials requiring prolonged observation of each patient. Br J Cancer.

[14] Cox DR (1972). Regression models and life tables. J R Stat Soc B.

[15] Li JX, Lu TX, Huang Y, Han F (2012). Clinical characteristics of recurrent nasopharyngeal carcinoma in high-incidence area. ScientificWorldJournal.

[16] Sun X, Su S, Chen C, Han F, Zhao C, Xiao W, Deng X, Huang S, Lin C, Lu T (2014). Long-term outcomes of intensity-modulated radiotherapy for 868 patients with nasopharyngeal carcinoma: an analysis of survival and treatment toxicities. Radiother Oncol.

[17] Xiao WW, Huang SM, Han F, Wu SX, Lu LX, Lin CG, Deng XW, Lu TX, Cui NJ, Zhao C (2011). Local control, survival, and late toxicities of locally advanced nasopharyngeal carcinoma treated by simultaneous modulated accelerated radiotherapy combined with cisplatin concurrent chemotherapy: long-term results of a phase 2 study. Cancer.

[18] Wang J, Shi M, Hsia Y, Luo S, Zhao L, Xu M, Xiao F, Fu X, Li J, Zhou B, Long X (2012). Failure patterns and survival in patients with nasopharyngeal carcinoma treated with intensity modulated radiation in Northwest China: a pilot study. Radiat Oncol.

[19] Osewski W, Dolla L, Radwan M, Szlag M, Rutkowski R, Smolinska B, Slosarek K (2014). Clinical examples of 3D dose distribution reconstruction, based on the actual MLC leaves movement, for dynamic treatment techniques. Rep Pract Oncol Radiother.

[20] Wan XB, Jiang R, Xie FY, Qi ZY, Li AJ, Ye WJ, Hua YJ, Zhu YL, Zou X, Guo L, Mai HQ, Guo X, Hong MH, Chen MY (2014). Endoscope-guided interstitial intensity-modulated brachytherapy and intracavitary brachytherapy as boost radiation for primary early T stage nasopharyngeal carcinoma. PLoS One.

[21] Ren YF, Gao YH, Cao XP, Ye WJ, Teh BS (2010). 3D-CT implanted interstitial brachytherapy for T2b nasopharyngeal carcinoma. Radiat Oncol.

[22] Leung TW, Tung SY, Wong VY, Sze WK, Lui CM, Wong FC, Lee AS, O.SK (2005). Nasopharyngeal intracavitary brachytherapy: the controversy of T2b disease. Cancer.

[23] Rosenblatt E, Abdel-Wahab M, El-Gantiry M, Elattar I, Bourque JM, Afiane M, Benjaafar N, Abubaker S, Chansilpa Y, Vikram B, Levendag P (2014). Brachytherapy boost in loco – regionally advanced nasopharyngeal carcinoma: a prospective randomized trial ofthe International Atomic Energy Agency. Radiat Oncol.

[24] Leibel SA, Scott CB, Mohiuddin M, Et A (1991). The effect of local-regional control on distant metastatic dissemination in carcinoma of the head and neck: results of an analysis from the RTOG head and neck database. Int J Radiat Oncol Biol Phys.

[25] Chang JT, Chan SC, Yen TC, Liao CT, Lin CY, Lin KJ, Chen IH, Wang HM, Chang YC, Chen TM, Kang CJ, Ng SH (2005). Nasopharyngeal carcinoma staging by (18) F-fluorodeoxyglucose positron emission tomography. Int J Radiat Oncol Biol Phys.

[26] Huang HI, Chan KT, Shu CH, Ho CY (2013). T4-locally advanced nasopharyngeal carcinoma: prognostic influence of cranial nerve involvement in different radiotherapy techniques. Sci World J.

[CR27] The pre-publication history for this paper can be accessed here: http://www.biomedcentral.com/1471-2407/14/894/prepub

